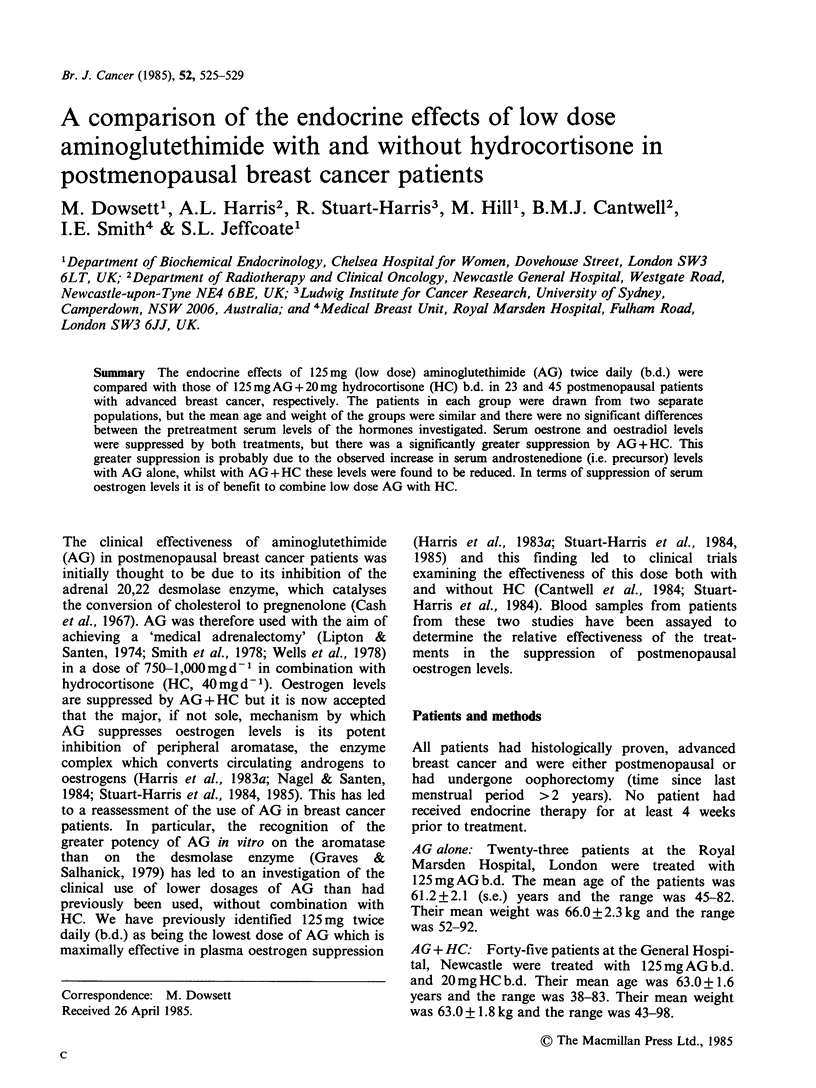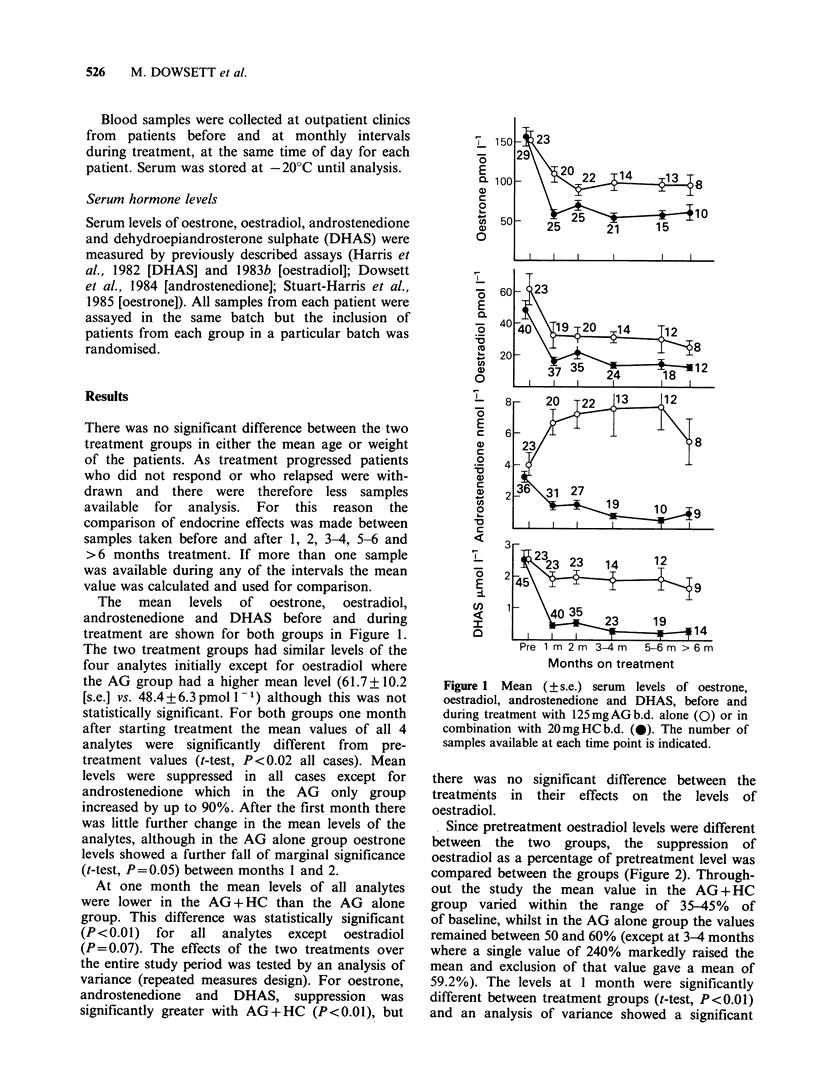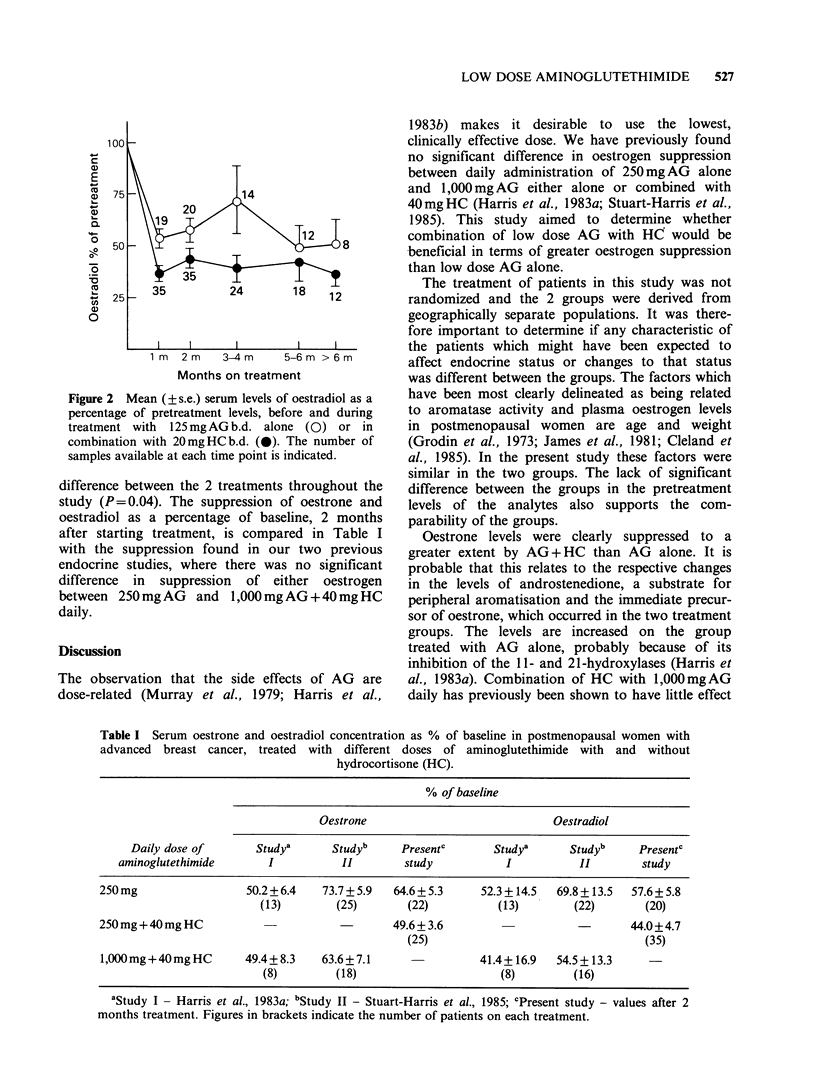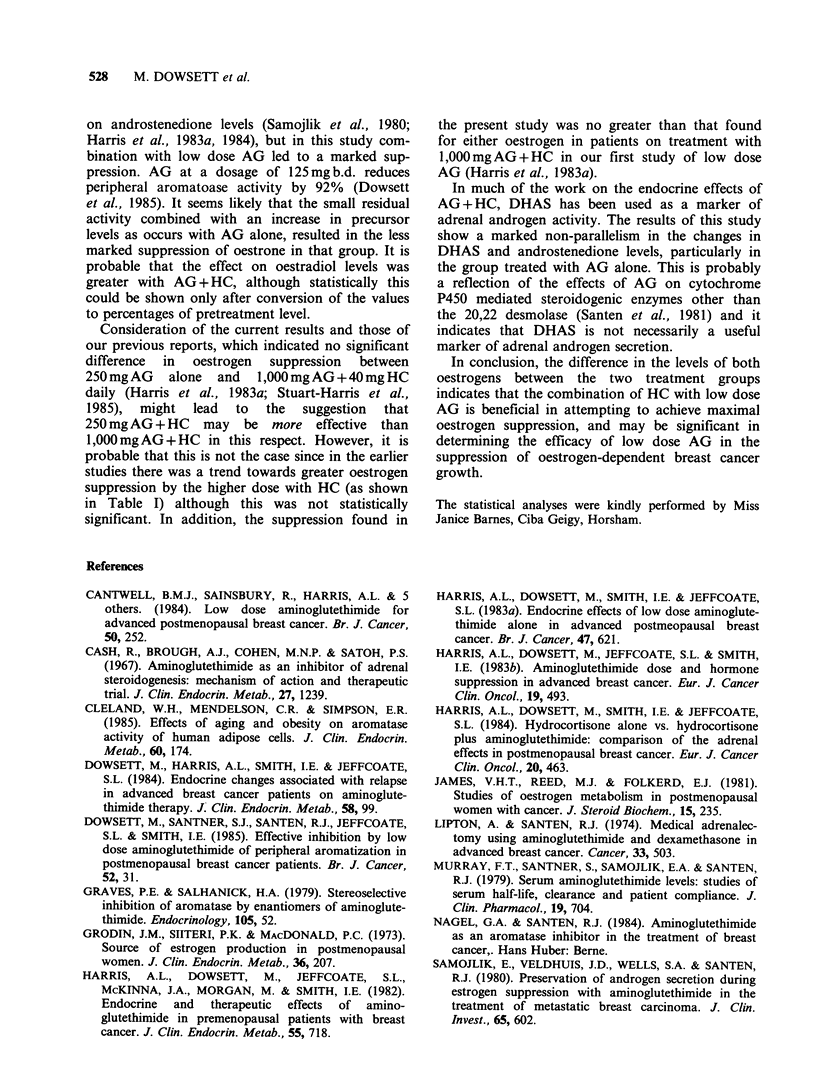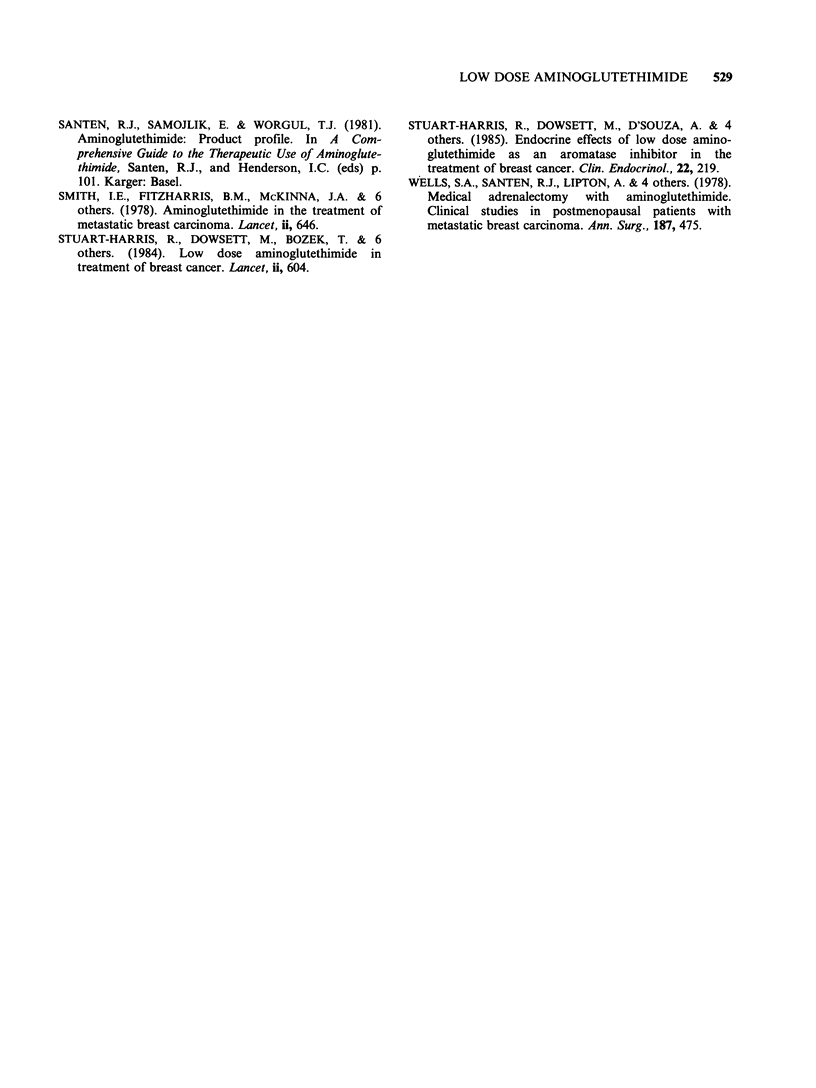# A comparison of the endocrine effects of low dose aminoglutethimide with and without hydrocortisone in postmenopausal breast cancer patients.

**DOI:** 10.1038/bjc.1985.223

**Published:** 1985-10

**Authors:** M. Dowsett, A. L. Harris, R. Stuart-Harris, M. Hill, B. M. Cantwell, I. E. Smith, S. L. Jeffcoate

## Abstract

The endocrine effects of 125 mg (low dose) aminoglutethimide (AG) twice daily (b.d.) were compared with those of 125 mg AG + 20 mg hydrocortisone (HC) b.d. in 23 and 45 postmenopausal patients with advanced breast cancer, respectively. The patients in each group were drawn from two separate populations, but the mean age and weight of the groups were similar and there were no significant differences between the pretreatment serum levels of the hormones investigated. Serum oestrone and oestradiol levels were suppressed by both treatments, but there was a significantly greater suppression by AG + HC. This greater suppression is probably due to the observed increase in serum androstenedione (i.e. precursor) levels with AG alone, whilst with AG + HC these levels were found to be reduced. In terms of suppression of serum oestrogen levels it is of benefit to combine low dose AG with HC.


*Br. J. Cancer* (1985), **52**, 525–529

### A comparison of the endocrine effects of low dose aminoglutethimide with and without hydrocortisone in postmenopausal breast cancer patients

M. Dowsett[1], A.L. Harris[2], R. Stuart-Harris[3], M. Hill[1], B.M.J. Cantwell[2], I.E. Smith[4] & S.L. Jeffcoate[1]

[1]*Department of Biochemical Endocrinology, Chelsea Hospital for Women, Dovehouse Street, London SW3 6LT, UK;* [2]*Department of Radiotherapy and Clinical Oncology, Newcastle General Hospital, Westgate Road, Newcastle-upon-Tyne NE4 6BE, UK;* [3]*Ludwig Institute for Cancer Research, University of Sydney, Camperdown, NSW 2006, Australia; and* [4]*Medical Breast Unit, Royal Marsden Hospital, Fulham Road, London SW3 6JJ, UK.*

**Summary** The endocrine effects of 125 mg (low dose) aminoglutethimide (AG) twice daily (b.d.) were compared with those of 125 mg AG + 20 mg hydrocortisone (HC) b.d. in 23 and 45 postmenopausal patients with advanced breast cancer, respectively. The patients in each group were drawn from two separate populations, but the mean age and weight of the groups were similar and there were no significant differences between the pretreatment serum levels of the hormones investigated. Serum oestrone and oestradiol levels were suppressed by both treatments, but there was a significantly greater suppression by AG + HC. This greater suppression is probably due to the observed increase in serum androstenedione (i.e. precursor) levels with AG alone, whilst with AG + HC these levels were found to be reduced. In terms of suppression of serum oestrogen levels it is of benefit to combine low dose AG with HC.

The clinical effectiveness of aminoglutethimide (AG) in postmenopausal breast cancer patients was initially thought to be due to its inhibition of the adrenal 20,22 desmolase enzyme, which catalyses the conversion of cholesterol to pregnenolone (Cash *et al.*, 1967). AG was therefore used with the aim of achieving a 'medical adrenalectomy' (Lipton & Santen, 1974; Smith *et al.*, 1978; Wells *et al.*, 1978) in a dose of 750–1,000 $mg\,d^{-1}$ in combination with hydrocortisone (HC, 40 $mg\,d^{-1}$). Oestrogen levels are suppressed by AG + HC but it is now accepted that the major, if not sole, mechanism by which AG suppresses oestrogen levels is its potent inhibition of peripheral aromatase, the enzyme complex which converts circulating androgens to oestrogens (Harris *et al.*, 1983*a*; Nagel & Santen, 1984; Stuart-Harris *et al.*, 1984, 1985). This has led to a reassessment of the use of AG in breast cancer patients. In particular, the recognition of the greater potency of AG *in vitro* on the aromatase than on the desmolase enzyme (Graves & Salhanick, 1979) has led to an investigation of the clinical use of lower dosages of AG than had previously been used, without combination with HC. We have previously identified 125 mg twice daily (b.d.) as being the lowest dose of AG which is maximally effective in plasma oestrogen suppression (Harris *et al.*, 1983*a*; Stuart-Harris *et al.*, 1984, 1985) and this finding led to clinical trials examining the effectiveness of this dose both with and without HC (Cantwell *et al.*, 1984; Stuart-Harris *et al.*, 1984). Blood samples from patients from these two studies have been assayed to determine the relative effectiveness of the treatments in the suppression of postmenopausal oestrogen levels.

Correspondence: M. Dowsett
Received 26 April 1985.

### Patients and methods

All patients had histologically proven, advanced breast cancer and were either postmenopausal or had undergone oophorectomy (time since last menstrual period >2 years). No patient had received endocrine therapy for at least 4 weeks prior to treatment.

*AG alone:* Twenty-three patients at the Royal Marsden Hospital, London were treated with 125 mg AG b.d. The mean age of the patients was 61.2 ± 2.1 (s.e.) years and the range was 45–82. Their mean weight was 66.0 ± 2.3 kg and the range was 52–92.

*AG + HC:* Forty-five patients at the General Hospital, Newcastle were treated with 125 mg AG b.d. and 20 mg HC b.d. Their mean age was 63.0 ± 1.6 years and the range was 38–83. Their mean weight was 63.0 ± 1.8 kg and the range was 43–98.

© The Macmillan Press Ltd., 1985

Blood samples were collected at outpatient clinics from patients before and at monthly intervals during treatment, at the same time of day for each patient. Serum was stored at $-20°C$ until analysis.

*Serum hormone levels*

Serum levels of oestrone, oestradiol, androstenedione and dehydroepiandrosterone sulphate (DHAS) were measured by previously described assays (Harris *et al.*, 1982 [DHAS] and 1983*b* [oestradiol]; Dowsett *et al.*, 1984 [androstenedione]; Stuart-Harris *et al.*, 1985 [oestrone]). All samples from each patient were assayed in the same batch but the inclusion of patients from each group in a particular batch was randomised.

### Results

There was no significant difference between the two treatment groups in either the mean age or weight of the patients. As treatment progressed patients who did not respond or who relapsed were withdrawn and there were therefore less samples available for analysis. For this reason the comparison of endocrine effects was made between samples taken before and after 1, 2, 3–4, 5–6 and >6 months treatment. If more than one sample was available during any of the intervals the mean value was calculated and used for comparison.

The mean levels of oestrone, oestradiol, androstenedione and DHAS before and during treatment are shown for both groups in Figure 1. The two treatment groups had similar levels of the four analytes initially except for oestradiol where the AG group had a higher mean level ($61.7 \pm 10.2$ [s.e.] *vs.* $48.4 \pm 6.3$ pmol $l^{-1}$) although this was not statistically significant. For both groups one month after starting treatment the mean values of all 4 analytes were significantly different from pre-treatment values (*t*-test, $P<0.02$ all cases). Mean levels were suppressed in all cases except for androstenedione which in the AG only group increased by up to 90%. After the first month there was little further change in the mean levels of the analytes, although in the AG alone group oestrone levels showed a further fall of marginal significance (*t*-test, $P=0.05$) between months 1 and 2.

At one month the mean levels of all analytes were lower in the AG+HC than the AG alone group. This difference was statistically significant ($P<0.01$) for all analytes except oestradiol ($P=0.07$). The effects of the two treatments over the entire study period was tested by an analysis of variance (repeated measures design). For oestrone, androstenedione and DHAS, suppression was significantly greater with AG+HC ($P<0.01$), but

**Figure 1** Mean (±s.e.) serum levels of oestrone, oestradiol, androstenedione and DHAS, before and during treatment with 125 mg AG b.d. alone (○) or in combination with 20 mg HC b.d. (●). The number of samples available at each time point is indicated.

there was no significant difference between the treatments in their effects on the levels of oestradiol.

Since pretreatment oestradiol levels were different between the two groups, the suppression of oestradiol as a percentage of pretreatment level was compared between the groups (Figure 2). Throughout the study the mean value in the AG+HC group varied within the range of 35–45% of of baseline, whilst in the AG alone group the values remained between 50 and 60% (except at 3–4 months where a single value of 240% markedly raised the mean and exclusion of that value gave a mean of 59.2%). The levels at 1 month were significantly different between treatment groups (*t*-test, $P<0.01$) and an analysis of variance showed a significant

**Figure 2** Mean (±s.e.) serum levels of oestradiol as a percentage of pretreatment levels, before and during treatment with 125 mg AG b.d. alone (○) or in combination with 20 mg HC b.d. (●). The number of samples available at each time point is indicated.

difference between the 2 treatments throughout the study ($P = 0.04$). The suppression of oestrone and oestradiol as a percentage of baseline, 2 months after starting treatment, is compared in Table I with the suppression found in our two previous endocrine studies, where there was no significant difference in suppression of either oestrogen between 250 mg AG and 1,000 mg AG + 40 mg HC daily.

### Discussion

The observation that the side effects of AG are dose-related (Murray *et al.*, 1979; Harris *et al.*, 1983*b*) makes it desirable to use the lowest, clinically effective dose. We have previously found no significant difference in oestrogen suppression between daily administration of 250 mg AG alone and 1,000 mg AG either alone or combined with 40 mg HC (Harris *et al.*, 1983*a*; Stuart-Harris *et al.*, 1985). This study aimed to determine whether combination of low dose AG with HC would be beneficial in terms of greater oestrogen suppression than low dose AG alone.

The treatment of patients in this study was not randomized and the 2 groups were derived from geographically separate populations. It was therefore important to determine if any characteristic of the patients which might have been expected to affect endocrine status or changes to that status was different between the groups. The factors which have been most clearly delineated as being related to aromatase activity and plasma oestrogen levels in postmenopausal women are age and weight (Grodin *et al.*, 1973; James *et al.*, 1981; Cleland *et al.*, 1985). In the present study these factors were similar in the two groups. The lack of significant difference between the groups in the pretreatment levels of the analytes also supports the comparability of the groups.

Oestrone levels were clearly suppressed to a greater extent by AG + HC than AG alone. It is probable that this relates to the respective changes in the levels of androstenedione, a substrate for peripheral aromatisation and the immediate precursor of oestrone, which occurred in the two treatment groups. The levels are increased on the group treated with AG alone, probably because of its inhibition of the 11- and 21-hydroxylases (Harris *et al.*, 1983*a*). Combination of HC with 1,000 mg AG daily has previously been shown to have little effect

**Table I** Serum oestrone and oestradiol concentration as % of baseline in postmenopausal women with advanced breast cancer, treated with different doses of aminoglutethimide with and without hydrocortisone (HC).

| | *% of baseline* | | | | | |
|---|---|---|---|---|---|---|
| | *Oestrone* | | | *Oestradiol* | | |
| *Daily dose of aminoglutethimide* | *Study*[a] *I* | *Study*[b] *II* | *Present*[c] *study* | *Study*[a] *I* | *Study*[b] *II* | *Present*[c] *study* |
| 250 mg | 50.2 ± 6.4 (13) | 73.7 ± 5.9 (25) | 64.6 ± 5.3 (22) | 52.3 ± 14.5 (13) | 69.8 ± 13.5 (22) | 57.6 ± 5.8 (20) |
| 250 mg + 40 mg HC | — | — | 49.6 ± 3.6 (25) | — | — | 44.0 ± 4.7 (35) |
| 1,000 mg + 40 mg HC | 49.4 ± 8.3 (8) | 63.6 ± 7.1 (18) | — | 41.4 ± 16.9 (8) | 54.5 ± 13.3 (16) | — |

[a]Study I – Harris *et al.*, 1983*a*; [b]Study II – Stuart-Harris *et al.*, 1985; [c]Present study – values after 2 months treatment. Figures in brackets indicate the number of patients on each treatment.

on androstenedione levels (Samojlik *et al.*, 1980; Harris *et al.*, 1983*a*, 1984), but in this study combination with low dose AG led to a marked suppression. AG at a dosage of 125 mg b.d. reduces peripheral aromatoase activity by 92% (Dowsett *et al.*, 1985). It seems likely that the small residual activity combined with an increase in precursor levels as occurs with AG alone, resulted in the less marked suppression of oestrone in that group. It is probable that the effect on oestradiol levels was greater with AG + HC, although statistically this could be shown only after conversion of the values to percentages of pretreatment level.

Consideration of the current results and those of our previous reports, which indicated no significant difference in oestrogen suppression between 250 mg AG alone and 1,000 mg AG + 40 mg HC daily (Harris *et al.*, 1983*a*; Stuart-Harris *et al.*, 1985), might lead to the suggestion that 250 mg AG + HC may be *more* effective than 1,000 mg AG + HC in this respect. However, it is probable that this is not the case since in the earlier studies there was a trend towards greater oestrogen suppression by the higher dose with HC (as shown in Table I) although this was not statistically significant. In addition, the suppression found in the present study was no greater than that found for either oestrogen in patients on treatment with 1,000 mg AG + HC in our first study of low dose AG (Harris *et al.*, 1983*a*).

In much of the work on the endocrine effects of AG + HC, DHAS has been used as a marker of adrenal androgen activity. The results of this study show a marked non-parallelism in the changes in DHAS and androstenedione levels, particularly in the group treated with AG alone. This is probably a reflection of the effects of AG on cytochrome P450 mediated steroidogenic enzymes other than the 20,22 desmolase (Santen *et al.*, 1981) and it indicates that DHAS is not necessarily a useful marker of adrenal androgen secretion.

In conclusion, the difference in the levels of both oestrogens between the two treatment groups indicates that the combination of HC with low dose AG is beneficial in attempting to achieve maximal oestrogen suppression, and may be significant in determining the efficacy of low dose AG in the suppression of oestrogen-dependent breast cancer growth.

The statistical analyses were kindly performed by Miss Janice Barnes, Ciba Geigy, Horsham.

### References

CANTWELL, B.M.J., SAINSBURY, R., HARRIS, A.L. & 5 others. (1984). Low dose aminoglutethimide for advanced postmenopausal breast cancer. *Br. J. Cancer*, **50**, 252.

CASH, R., BROUGH, A.J., COHEN, M.N.P. & SATOH, P.S. (1967). Aminoglutethimide as an inhibitor of adrenal steroidogenesis: mechanism of action and therapeutic trial. *J. Clin. Endocrin. Metab.*, **27**, 1239.

CLELAND, W.H., MENDELSON, C.R. & SIMPSON, E.R. (1985). Effects of aging and obesity on aromatase activity of human adipose cells. *J. Clin. Endocrin. Metab.*, **60**, 174.

DOWSETT, M., HARRIS, A.L., SMITH, I.E. & JEFFCOATE, S.L. (1984). Endocrine changes associated with relapse in advanced breast cancer patients on aminoglutethimide therapy. *J. Clin. Endocrin. Metab.*, **58**, 99.

DOWSETT, M., SANTNER, S.J., SANTEN, R.J., JEFFCOATE, S.L. & SMITH, I.E. (1985). Effective inhibition by low dose aminoglutethimide of peripheral aromatization in postmenopausal breast cancer patients. *Br. J. Cancer*, **52**, 31.

GRAVES, P.E. & SALHANICK, H.A. (1979). Stereoselective inhibition of aromatase by enantiomers of aminoglutethimide. *Endocrinology*, **105**, 52.

GRODIN, J.M., SIITERI, P.K. & MACDONALD, P.C. (1973). Source of estrogen production in postmenopausal women. *J. Clin. Endocrin. Metab.*, **36**, 207.

HARRIS, A.L., DOWSETT, M., JEFFCOATE, S.L., MCKINNA, J.A., MORGAN, M. & SMITH, I.E. (1982). Endocrine and therapeutic effects of aminoglutethimide in premenopausal patients with breast cancer. *J. Clin. Endocrin. Metab.*, **55**, 718.

HARRIS, A.L., DOWSETT, M., SMITH, I.E. & JEFFCOATE, S.L. (1983*a*). Endocrine effects of low dose aminoglutethimide alone in advanced postmeopausal breast cancer. *Br. J. Cancer*, **47**, 621.

HARRIS, A.L., DOWSETT, M., JEFFCOATE, S.L. & SMITH, I.E. (1983*b*). Aminoglutethimide dose and hormone suppression in advanced breast cancer. *Eur. J. Cancer Clin. Oncol.*, **19**, 493.

HARRIS, A.L., DOWSETT, M., SMITH, I.E. & JEFFCOATE, S.L. (1984). Hydrocortisone alone vs. hydrocortisone plus aminoglutethimide: comparison of the adrenal effects in postmenopausal breast cancer. *Eur. J. Cancer Clin. Oncol.*, **20**, 463.

JAMES, V.H.T., REED, M.J. & FOLKERD, E.J. (1981). Studies of oestrogen metabolism in postmenopausal women with cancer. *J. Steroid Biochem.*, **15**, 235.

LIPTON, A. & SANTEN, R.J. (1974). Medical adrenalectomy using aminoglutethimide and dexamethasone in advanced breast cancer. *Cancer*, **33**, 503.

MURRAY, F.T., SANTNER, S., SAMOJLIK, E.A. & SANTEN, R.J. (1979). Serum aminoglutethimide levels: studies of serum half-life, clearance and patient compliance. *J. Clin. Pharmacol.*, **19**, 704.

NAGEL, G.A. & SANTEN, R.J. (1984). Aminoglutethimide as an aromatase inhibitor in the treatment of breast cancer,. Hans Huber: Berne.

SAMOJLIK, E., VELDHUIS, J.D., WELLS, S.A. & SANTEN, R.J. (1980). Preservation of androgen secretion during estrogen suppression with aminoglutethimide in the treatment of metastatic breast carcinoma. *J. Clin. Invest.*, **65**, 602.

SANTEN, R.J., SAMOJLIK, E. & WORGUL, T.J. (1981). Aminoglutethimide: Product profile. In *A Comprehensive Guide to the Therapeutic Use of Aminoglutethimide,* Santen, R.J., and Henderson, I.C. (eds) p. 101. Karger: Basel.

SMITH, I.E., FITZHARRIS, B.M., MCKINNA, J.A. & 6 others. (1978). Aminoglutethimide in the treatment of metastatic breast carcinoma. *Lancet,* **ii,** 646.

STUART-HARRIS, R., DOWSETT, M., BOZEK, T. & 6 others. (1984). Low dose aminoglutethimide in treatment of breast cancer. *Lancet,* **ii,** 604.

STUART-HARRIS, R., DOWSETT, M., D'SOUZA, A. & 4 others. (1985). Endocrine effects of low dose aminoglutethimide as an aromatase inhibitor in the treatment of breast cancer. *Clin. Endocrinol.,* **22,** 219.

WELLS, S.A., SANTEN, R.J., LIPTON, A. & 4 others. (1978). Medical adrenalectomy with aminoglutethimide. Clinical studies in postmenopausal patients with metastatic breast carcinoma. *Ann. Surg.,* **187,** 475.